# Development of a predictive machine learning model for pathogen profiles in patients with secondary immunodeficiency

**DOI:** 10.1186/s12911-024-02447-w

**Published:** 2024-02-13

**Authors:** Qianning Liu, Yifan Chen, Peng Xie, Ying Luo, Buxuan Wang, Yuanxi Meng, Jiaqian Zhong, Jiaqi Mei, Wei Zou

**Affiliations:** 1 School of Statistics, Jiangxi University of Finance and Economics, Nanchang, 330013 Jiangxi China; 2 Department of Infectious Diseases, The First Affiliated Hospital, Jiangxi Medical College, Nanchang University, Nanchang, 330006 Jiangxi China; 3 The First Clinical Medical College,Jiangxi Medical College, Nanchang University, Nanchang, 330006 Jiangxi China; 4 Department of Infectious Diseases, Third People’s Hospital of Jiujiang, Jiujiang, 332000 Jiangxi China

**Keywords:** Secondary immunodeficiency, Pathogens, Imbalanced data, K nearest neighbour, Boosted logistic regression, Random forest, Gradient boosting machine

## Abstract

**Background:**

Secondary immunodeficiency can arise from various clinical conditions that include HIV infection, chronic diseases, malignancy and long-term use of immunosuppressives, which makes the suffering patients susceptible to all types of pathogenic infections. Other than HIV infection, the possible pathogen profiles in other aetiology-induced secondary immunodeficiency are largely unknown.

**Methods:**

Medical records of the patients with secondary immunodeficiency caused by various aetiologies were collected from the First Affiliated Hospital of Nanchang University, China. Based on these records, models were developed with the machine learning method to predict the potential infectious pathogens that may inflict the patients with secondary immunodeficiency caused by various disease conditions other than HIV infection.

**Results:**

Several metrics were used to evaluate the models’ performance. A consistent conclusion can be drawn from all the metrics that Gradient Boosting Machine had the best performance with the highest accuracy at 91.01%, exceeding other models by 13.48, 7.14, and 4.49% respectively.

**Conclusions:**

The models developed in our study enable the prediction of potential infectious pathogens that may affect the patients with secondary immunodeficiency caused by various aetiologies except for HIV infection, which will help clinicians make a timely decision on antibiotic use before microorganism culture results return.

## Introduction

Human immune system plays a crucial role against all kinds of pathogens [1]. Defects in any components of the immune system lead to immunodeficiency. Depending on the underlying mechanisms, immunodeficiency is categorized as primary and secondary [2]. Primary immunodeficiency often involves genetic mutations in the components of immune system and includes antibody deficiency, complement deficiency, phagocytic deficiency and combined immune deficiency et al. [3–5]. Secondary immunodeficiency, which is the topic of our current study, can occur in the circumstances of human immunodeficiency virus (HIV) infection, long-term use of immunosuppressives, severe burns, chronic kidney diseases and malignant tumors et al. [6–8].

In HIV-infected patients the spectrum of pathogens causing opportunistic infections is well-documented, with specific reference to the CD4+ T cell counts influencing susceptibility to various pathogens [9–13]. However, the pathogen profiles responsible for the opportunistic infections in individuals with other forms of secondary immunodeficiency remain largely unexplored [14, 15]. While blood or other sterile body fluid culture remains the gold standard for diagnosing infections [16–18], it has certain limitations such as time-consuming and a low positive rate [19]. Therefore, development of other methods capable of informing potential pathogens in patients with secondary immunodeficiency is crucial for timely guiding clinical decision-making, which sometimes is life-saving.

Mathematical models are valuable tools for infectious disease research [20], and one of their most important applications is predicting disease occurrence. With the development of different mathematical models postoperative infection in elderly spinal fractures [21], high risk types of human papillomaviruse infection [22] and disease outcome of septic shock [23] can be predicted. However, mathematical models haven’t yet been applied to predict the pathogen profiles commonly seen in the secondary immunodeficiency other than HIV infection. It is actually an important question considering the huge size of this patient population and the time it takes for the standard microbiological methods to report positive results. Therefore, to facilitate timely diagnosis of infections in patients with secondary immunodeficiency caused by the etiologies other than HIV, in the current study we intend to construct mathematical models that are based on certain lab test results including complete blood count (CBC), C-reactive protein (CRP), procalcitonin (PCT), erythrocyte sedimentation rate (ESR) and culture results from various body fluids, to help clinicians predict the most likely pathogens and then rapidly start empirical antibiotics before the culture results return in this patient population.

## Methods

### Data collection

Our study was approved by the ethical committee of The First Affiliated Hospital of Nanchang University with reference number (2022) CDYFYYLK (10–010). Data were collected from the medical records of the patients from the department of hematology, transplantation ICU, autoimmune diseases, oncology, intensive care unit (ICU), nephrology and burn from the year of 2012 to 2022.

Based on the definition of the non HIV infected immunodeficient population in the “Clinical practice guideline on early detection for pulmonary tuberculosis in general hospitals” that was published in China in late 2023 [24], all the included patients had infections of blood, abdominal cavity or other body locations that were secondary to immunodeficiency caused by either their original diseases or the treatments they received. Specimen was collected for culture from the infection sites once infection was suspicious.

The patient inclusion criteria of current study include: 1. Patients with hematological malignancies undergoing chemotherapy and/or radiology; 2. Patients with solid malignant tumors undergoing chemotherapy, radiology and/or surgery with bone marrow suppression for more than 2 weeks; 3. Patients with rheumatoid autoimmune diseases on long-term use of glucocorticoids (defined as prednisone≥30 mg/d or equivalent dosage of prednisone at 0.5 mg/kg/day for more than 2 weeks) and/or cytotoxic drugs; 4. Patients with hypoproteinemia caused by organ dysfunction such as cirrhosis and liver failure or protein loss due to chronic kidney disease; 5. Patients after organ transplantation on immunosuppressants; 6. Patients with severe burn; 7. Patients in ICU with possible secondary immunodeficiency. The exclusion criteria include:1. Short-term use of immunosuppressants (less than 2 weeks); 2. Primary immunodeficiency; 3. People living with HIV; 4. Patients with secondary immunodeficiency whose body fluid culture turned out to be negative; 5. Patients with secondary immunodeficiency whose culture results were suspicious of contamination.

### Data preprocessing

The data containing 2024 observations and 42 features were collected from the medical records of the 1st affiliated hospital of Nanchang University in China from 2012 to 2022. Among these data, 1053 pieces were from the department of hematology, 13 pieces were from transplantation ICU, 114 pieces were from the department of autoimmune diseases, 104 pieces were from the department of oncology, 109 pieces were from ICU, 213 pieces were from the department of nephrology and 418 pieces of them were from the department of burn. The original dataset contains a great amount of missing information, and the data matrix is sparse with a lot of tedious text information thus needing to be preprocessed.

To ensure the models’ effectiveness, we deleted the records with missing values while retaining the features such as PCT and CRP that were significantly associated with cultivation results. After preprocessing, the data matrix had 443 observations and 13 columns left.

Finally, we had to deal with unstructured data. The column of cultivation results, as the response variable, needed to be first classified manually to prevent the reduction of model efficiency caused by too many categories. We realized that the number of manual classifications should not be too many or few. On one hand, if the number of categories was too few and in consideration of there being only two classes, Gram (+) and Gram (−) bacterial, our study would have no medical meaning. On the other hand, if the number of categories was too many, for example, 50 small classes, the effect of the model would be foreseeably terrible. Therefore, we categorized the cultivation results into seven classes based on the genus of the bacteria.

However, other text features like “Diagnostic Results” and “Infection Sites” could not be dealt with this way. The reason is that we could not easily classify them into different categories. For example, a patient was diagnosed with leukemia and diabetes, but we could not categorize it into a class called “Leukemia and Diabetes”, as the classes would be too miscellaneous in this way. A better approach would be transforming these two features into dummy variables. We used the tidyverse package in R to pull out the critical information in these two columns and create the dummy variables.

### Imbalanced class

However, one question still remained. The seven classes in response variables were highly imbalanced, which may cause serious problems. For instance, the classifier would tend to categorize a small class into a big one although it still had acceptable performance. Although under-sampling and oversampling are the most widely used approaches to address this imbalance problem, they are also likely to cause underfitting and overfitting, respectively. In our study, we chose multinomial distribution sampling, which was proved feasible in the application [25], to tackle the imbalanced problem. The seven classes were resampled according to a multinomial distribution with probability {*q*_*i*_}_*i* = 1…7_, where:

$$q_{i}=\frac{p_{i}^{\alpha}}{\sum_{j=1}^7 p_{j}^{\alpha}}\ {with}\ {p}_{i}=\frac{n_{i}}{\sum_{k=1}^7n_{k}}$$

*α* = 0.5 was employed in our study. Sampling with this distribution would increase the number of small classes and decrease the number of big classes but not cause severe information loss and overfitting problems.

### Feature selection

After transforming the “Diagnostic Results” and “Infection Sites” columns into dummy variables, there were 88 features in total. However, too many variables in the model will cause multicollinearity and overfitting. Accordingly, to simplify the model and retain the model performance, we need to reduce the dimensions. We choose the Recursive Feature Elimination method to perform the feature selection and include the top 20 most important variables in our model. Among these variables, PCT level, absolute numbers of lymphocytes and leukocytes, percentage of neutrocytes, age, number of neutrocytes, CRP, bone marrow suppression, lung infection, gender, septicemia and leukemia are the top 12 important variables.

### Models

Our primary goal is to predict the cultivation results. Therefore, we applied several machine learning models to the data and observed their performance. After completing initial data preprocessing steps including missing data handling, feature extraction, feature selection, and outcome weighting, the dataset was split into training and testing sets with an 80:20 ratio. The training set was used to develop and tune the prediction models, while the testing set was held out solely for final model evaluation.

Our first model, K Nearest Neighbor (KNN), was correlated with Euclidean distance. The only hyperparameter of this algorithm was the number of the nearest neighbors, k. In order to select the best hyperparameter, we used grid search to iterate k through 1 to 20.

Boosted logistic regression also had only one hyperparameter, nIter, representing the number of boosting iterations. We also set the range of nIter from 1 to 30 to perform the grid search.

Random Forest that was trained by the bagging method is an ensemble algorithm of Decision Trees. It also had only one hyperparameter, mtry. We also used grid search to iterate mtry, which represented the number of randomly selected predictors. The mtry value set was *mtry* = {2, 3, 4, 5, 6, 7, 8, 9, 10,12,14,16,18,20,25,30,35,40,45,50}.

Gradient Boosting Machine is an ensemble of weak learners which has several hyperparameters. Since the number of parameters was greater than one, we used grid search to explore all the possible combinations till we reached a set that generated the best performance. This set included max tree depth {1, 5, 10,15,20} and number of trees {50,100,150,200, …, 450,500}.

### Model selection

We used grid search to explore the best hyperparameters to obtain the best model performance. Grid search is a popular model selection method that iterates through every possible parameter combination to find the best one. The process is mechanized and effective.

**Fig. 1:**
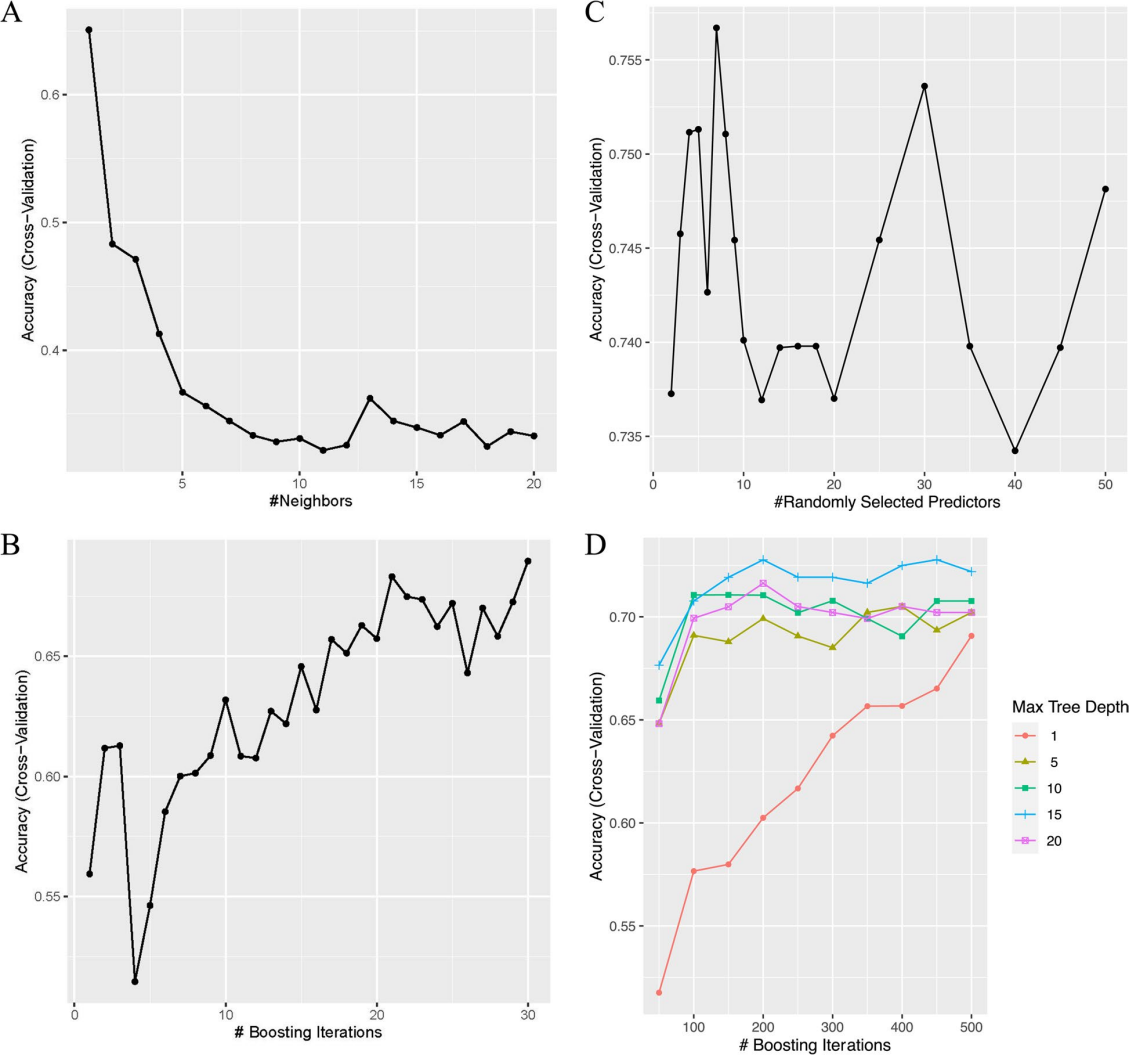
Hyperparameter Optimization Across Different Machine Learning Models. **A**-**D** Performance metrics obtained from a 10-fold cross-validation grid search. **A** K-Nearest Neighbors (KNN) model accuracy as a function of the number of neighbors, with optimal performance at k = 1. **B** Boosted Logistic Regression model accuracy across boosting iterations, peaking at nIter = 30. **C** Random Forest model accuracy in relation to the number of randomly selected predictors, optimal at mtry = 6. **D** Gradient Boosting Machine model accuracy influenced by the number of boosting iterations and maximum tree depth, with the highest accuracy achieved at a depth of 15 and 450 trees. The selection of k = 1 for the KNN model suggests a potential overfitting issue that warrants further evaluation

In Fig. 1, panels A-D depict the results of a 10-fold cross-validation grid search on the training dataset. Panel A details the KNN model, where the optimal number of neighbors (k) is 1. Panel B illustrates the Boosted Logistic Regression model, with the best performance at 30 boosting iterations (nIter). Panel C shows the Random Forest model, achieving highest accuracy with 6 randomly selected predictors (mtry). Finally, Panel D presents the Gradient Boosting Machine, where a maximum tree depth of 15 and 450 trees resulted in the highest accuracy. Notably, the optimal parameter for KNN, k = 1, raises concerns regarding potential overfitting.

### Metrics

#### Adjustment on multiclass ROC curve

Receiver Operating Characteristic Curve, also known as ROC Curve, is widely used for assessing binary classification problems. Our problem, however, is related to multiclass classification. Thus, the ROC curve needs some adjustments. There are two ways to address this problem.

- Let *n* be the number of samples in the testing set, and $\mathcal{P}$ be the number of classes. After training the model in the training set, we generated a probability matrix with *n* rows and $\mathcal{P}$ columns where the (*i*, *j*) entry represented the probability of the *i*^*th*^ sample in the *j*^*th*^ class. Under every category, we could plot a ROC curve. Therefore, we could get $\mathcal{P}$ ROC curves in the end. After calculation of their mean, we would have the final ROC curve.
- Create a label matrix that has the same structure as the probability matrix. Each row is a sample’s one-hot code representing the class’s label. By converting these two matrices into two vectors by column and transposing them respectively, we got two column vectors which could be seen as the probability matrix of binary classification. Then we can easily plot the ROC curve.

**Fig. 2:**
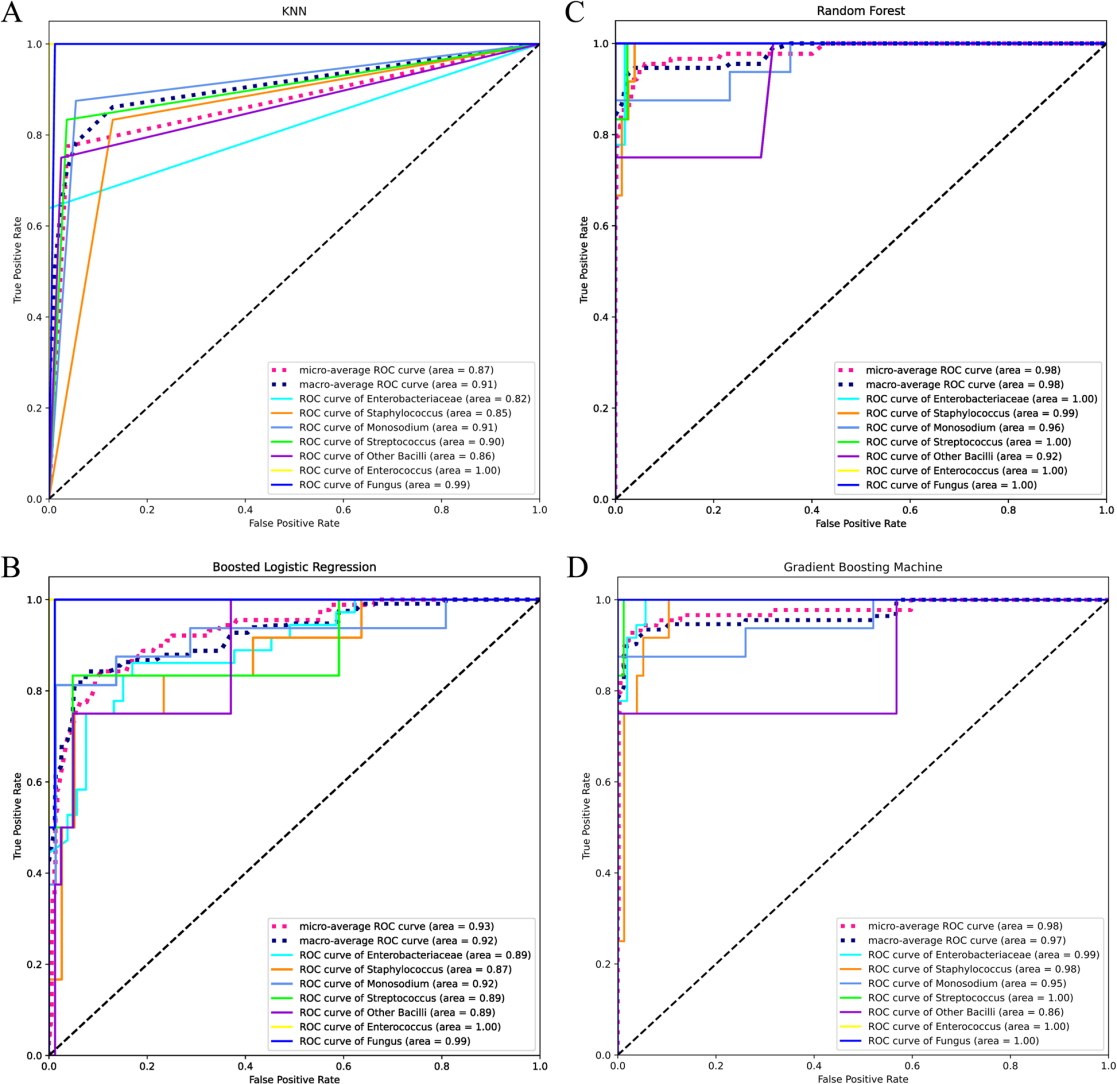
Receiver Operating Characteristic (ROC) Curves for Machine Learning Models. **A**-**D** Comparison of model performance through ROC analysis. **A** K-Nearest Neighbors (KNN) ROC curves for various classes, highlighting the trade-off between true positive rate and false positive rate with an area under the curve (AUC) for the micro-average at 0.87 and macro-average at 0.91. **B** Boosted Logistic Regression ROC curves, showing improved performance with an AUC for the micro-average at 0.93 and macro-average at 0.92. **C** Random Forest ROC curves, indicating superior performance with an AUC for the micro-average at 0.98 and macro-average at 0.98. **D** Gradient Boosting Machine ROC curves, exhibiting exceptional discriminative power with an AUC for the micro-average at 0.98 and macro-average at 0.97

The above two approaches are ‘macro’ and ‘micro’ scenarios in the sklearn.metric.roc_auc_score() function. The results of these two different methods should be close to each other. Therefore, we included both of them in our Fig. 2 to ensure the robustness of the models.

Figure 2A-D display ROC curves for K-Nearest Neighbors, Boosted Logistic Regression, Random Forest, and Gradient Boosting Machine models, delineating their discriminative performance in pathogen classification, with the latter two models showing notably superior performance as evidenced by their near-perfect micro-average and macro-average AUCs of 0.98.

#### Confusion matrix

The confusion matrix is a specific table layout that visualizes the performance of the classification model. In the matrix, each column represents the true value, and each row represents the predicted value. Typically, the bigger the values in the diagonal are, the better the model performs.

**Fig. 3:**
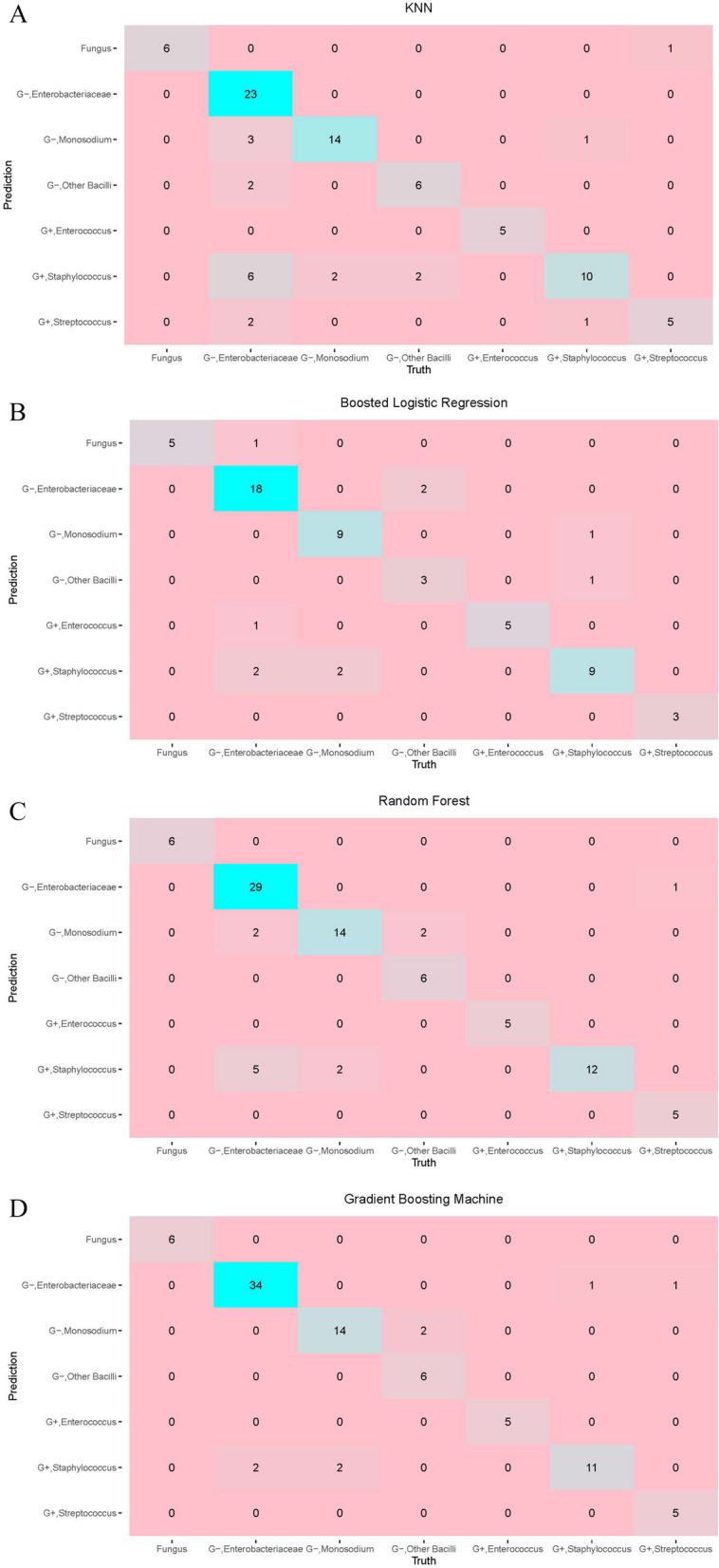
Confusion Matrices for Model Performance Evaluation. This figure presents the confusion matrices of four machine learning models, allowing for a detailed assessment of prediction accuracy for various pathogen classes. Values along the diagonal represent correct classifications, while non-diagonal values indicate misclassifications. **A** The K-Nearest Neighbors (KNN) model matrix, with counts of true vs. predicted labels, showing a specific number for true positive rates in pathogen detection. **B** The Boosted Logistic Regression model matrix, detailing the true positives along the diagonal and misclassifications off-diagonal, reflecting the model’s predictive power and misclassification patterns. **C** The Random Forest model matrix, which illustrates a higher concentration of true positives along the diagonal, indicative of a model with strong predictive accuracy. **D** The Gradient Boosting Machine model matrix, showing high true positive rates, especially for certain pathogens, suggesting a high degree of model precision

Figure 3A-D depict confusion matrices for the K-Nearest Neighbors, Boosted Logistic Regression, Random Forest, and Gradient Boosting Machine models, respectively, illustrating their classification accuracy across various pathogens. Each matrix shows the number of correct and incorrect predictions, with darker shades indicating higher counts. The Gradient Boosting Machine demonstrates the highest number of correct predictions for Enterobacteriaceae, while all models show good performance in correctly identifying the majority of pathogens, albeit with some false positives and negatives, as is typical in predictive modeling.

#### Accuracy, recall, precision, brier score and F1 score

Accuracy is the most popular metric in classification problems. However, it may not be the most appropriate one for this issue because of the remaining imbalanced classes. We also used recall, precision, Brier Score, and F1-score as metrics. Notice that there are a few differences in these metrics between multiclass and binary classifications. True positive (TP) is just the entry that correctly predicts the true category as usual. However, false-negative (FN) is the sum of the whole row in the confusion matrix except true positive ones. Likewise, false positive (FP) is the sum of the whole columns except true positive elements. Apart from that, all the definitions are the same as those in binary classification.

$${Accuracy}=\frac{{TP}+{TN}}{{TP}+{TN}+{FP}+{FN}}$$

$${Recall}=\frac{{TP}}{{TP}+{FN}}$$

$${Precision}=\frac{{TP}}{{TP}+{FP}}$$

$${{F}}_1=\frac{2}{\frac{1}{{Recall}}+\frac{1}{{Precision}}}$$

## Results

### Descriptive statistics

Demographic characteristics and infection-associated lab test results of studied patients from the department of hematology, rheumatology, oncology, ICU, nephrology and burn were shown in Table 1. Except for the rheumatology department, male patients out-numbered female patients in all the other departments. The ages of male and female patients were compatible within each department. Patients from ICU were the oldest, followed by oncology department, nephrology department, rheumatology department, hematology department and burn department in age. Only hematology, oncology and burn department have collected enough data of CRP and PCT for analysis. As seen from Table 1, CRP and PCT of the patients from these 3 departments were dramatically elevated. Except for the patients from hematology and rheumatology department, and the female patients from oncology department, all the other patients had elevated blood leukocyte number. Normal to slightly elevated leukocytes in the patients from hematology and rheumatology department and in the female patients from oncology department may be partly related to other treatments they received such as immunosuppressive therapy and chemotherapy. The absolute number of peripheral neutrocytes in all the patients was elevated. Interestingly, the absolute number of peripheral lymphocytes in the patients from hematology department was dramatically elevated but that in the patients from all the other departments was normal, which probably was related to the original diseases that the patients from hematology department were suffering.

**Table 1:** Demographic characteristics and infection-associated lab test results of studied patients

		Age	CRP	PCT	Leukocytes	Neutrocytes	Lymphocytes
Hematology Department	Male 54.16%	40.31	105.03	17.90	9.23	8.31	11.06
	Female 45.84%	44.36	105.22	16.65	4.62	7.92	12.08
Rheumatology Department	Male 36.28%	53.41	NA	NA	9.73	6.44	2.20
	Female 63.72%	47.94	NA	NA	9.69	7.16	1.60
Oncology Department	Male 65.71%	59.69	105.63*	17.67*	10.07	8.05	1.26
	Female 34.29%	54.61	195.34*	12.17*	9.17	6.96	1.39
ICU	Male 63.89%	61.09	NA	NA	12.87	6.75	2.01
	Female 36.11%	55.79	NA	NA	9.95	7.26	1.33
Nephrology Department	Male 63.55%	52.92	NA	NA	12.35	9.29	1.38
	Female 36.45%	59.86	NA	NA	10.9	8.18	1.99
Burn Department	Male 69.69%	36.97	124.06*	18.05*	13.58	9.77	1.89
	Female 30.31%	32.4	115.43*	8.87*	13.16	9.77	2.11

^*^The number of non-NA values is relatively too few to make a reliable estimation

Other interesting information that serves great medical purposes can further be extracted from Table 1. The number of white blood cells in males has more extreme values than in females. That is to say, there are many male patients whose number of leukocytes is a lot greater than 100 × 10^9^∕ while the greatest value in females is 99.38 × 10^9^ ∕ *L*, which probably is associated with the biological difference between men and women. When it comes to infection location, the characteristics of blood infection is very unique compared to those of other infection locations. First, the mean PCT of blood infection patients is 78.94*ng* ∕ *ml* and the mean number of lymphocytes is 45.03 × 10^9^ ∕ *L*, which are both much higher than those in other infection locations. This observation is consistent with what has been known about the clinical significance of PCT. In general, PCT increases slightly in patients with local bacterial infection but increases significantly among those with invasive infection such as blood infection causing systemic inflammatory response syndrome (SIRS), sepsis and septic shock [19]. Then, the mean number of neutrocyte in males with blood infection is 40.71 × 10^9^ ∕ *L*, which is much higher compared to the others. In the end, the mean CRP in blood infection patients is around 0 and it is much lower than the others, indicating its specificity and sensitivity may be lower than PCT.

### Model evaluation

As seen in Fig. 2, KNN and Boosted Logistic Regression didn’t perform as well as Random Forest and Gradient Boosting Machine. It may be a shred of partial evidence that ensemble algorithms are more appropriate for this dataset. However, we still need further evidence to verify it.

As shown in Fig. 3, KNN has a significant misclassification. Although the confusion matrix gave us so much specific information about models’ performance on the test set, it could hardly be called a straightforward and quantified metric because it could neither directly tell models’ specific performance nor easily be compared to other models. Therefore, we need to employ some quantified metrics to identify the performance of each model and make a comparison between different models.

The ensemble algorithms, Random Forest and Gradient Boosting Machine exceed others in performance. Meanwhile, we analyzed this table along with the confusion matrix and found some interesting facts. KNN and Random Forest tended to misclassify Enterobacteriaceae into other bacteria including Pseudomonas and Staphylococcus. Moreover, all these four classifiers seemed not good at predicting Staphylococcus and tended to misclassify other bacteria into Staphylococcus since they all had a relatively low precision score.

Table 2 presents a comprehensive overview of the performance metrics for four different machine learning models used to predict the presence of various pathogens in a clinical setting. The performance of each model is evaluated in terms of Accuracy, Brier Score, Precision, Recall, and F1 Score across different bacteria, as well as fungal infections, which provides a robust set of criteria for assessing the predictive capabilities.

**Table 2:** Performance of the models

Models	Bacteria	Accuracy	Brier Score	Precision	Recall	F1 Score
KNN	Enterobacteriaceae	0.775 (0.674, 0.857)*	0.449	1.000	0.638	0.779
	Staphylococcus			0.500	0.833	0.625
	Monosodium			0.777	0.875	0.823
	Streptococcus			0.625	0.833	0.714
	Other Bacilli			0.750	0.750	0.750
	Enterococcus			1.000	1.000	1.000
	Fungus			0.857	1.000	0.923
Boosted Logistic Regression	Enterobacteriaceae	0.838 (0.723, 0.919)*	0.460	0.900	0.818	0.857
	Staphylococcus			0.692	0.818	0.750
	Monosodium			0.900	0.818	0.857
	Streptococcus			1.000	1.000	1.000
	Other Bacilli			0.750	0.600	0.666
	Enterococcus			0.833	1.000	0.909
	Fungus			0.833	1.000	0.909
Random Forest	Enterobacteriaceae	0.865 (0.776, 0.928)*	0.221	0.966	0.805	0.878
	Staphylococcus			0.631	1.000	0.774
	Monosodium			0.777	0.875	0.823
	Streptococcus			1.000	0.833	0.909
	Other Bacilli			1.000	0.750	0.857
	Enterococcus			1.000	1.000	1.000
	Fungus			1.000	1.000	1.000
Gradient Boosting Machine	Enterobacteriaceae	0.910 (0.830, 0.960)*	0.214	0.944	0.944	0.944
	Staphylococcus			0.733	0.916	0.814
	Monosodium			0.875	0.875	0.875
	Streptococcus			1.000	0.833	0.909
	Other Bacilli			1.000	0.750	0.857
	Enterococcus			1.000	1.000	1.000
	Fungus			1.000	1.000	1.000

^*^The 95% confidence interval of accuracy

The KNN model shows consistent performance, especially for the Enterobacteriaceae genus, with an accuracy of 77.53%, complemented by a moderate Brier Score of 0.449, and a high F1 Score of 0.779 due to its perfect precision. It also performs commendably well with fungi, achieving an F1 Score of 0.923 with a Brier Score that indicates reliable probabilistic predictions.

The Boosted Logistic Regression model overall appears to provide strong results, especially with the Streptococcus genus for which it achieves perfect scores in all three metrics, along with a Brier Score that supports the reliability of its probabilistic assessments. Furthermore, the model maintains high predictive power for both Enterobacteriaceae and fungi, with accuracy rates surpassing 83% and Brier Scores that reflect the consistency of the model’s predictions.

The Random Forest model exhibits outstanding performance, with exceptional results for the Streptococcus and Enterococcus genera, along with fungi – each achieving perfect accuracy, precision, and F1 Scores of 1.0000. These results are further corroborated by the model’s low Brier Scores, particularly noteworthy being the 0.221 for Enterobacteriaceae, indicating the model’s robust ability to correctly classify and balance the presence of these pathogens within the dataset with reliable probability estimates.

The Gradient Boosting Machine (GBM) model demonstrates exemplary performance overall, particularly with the Enterobacteriaceae genus, boasting an accuracy of 91.01% and a corresponding F1 Score of 0.944. The Brier Score of 0.214 for this model indicates a high level of precision in the probabilistic predictions, complementing its exceptional ability to balance precision and recall. It also performs flawlessly for Streptococcus, other Bacilli, Enterococcus, and fungi, as reflected by a perfect F1 Score of 1.0000 and supportive Brier Scores indicating accurate probability estimates.

The confidence intervals for accuracy, along with the Brier Scores, are provided for each genus within the models, which allows for a comprehensive statistical understanding of the accuracy and reliability of the predictions. Notably, for pathogens with the higher prevalence, the models demonstrate tight confidence intervals and lower Brier Scores, implicating robustness in their predictive accuracy.

In conclusion, this comparative analysis illustrates that advanced machine learning techniques can be highly effective for pathogen prediction in patients with secondary immunodeficiency.

## Discussion

Many non-infectious diseases have as a common complication the secondary development of infections, which more often occurs in the circumstances of long-term use of immunosuppressives, severe trauma, malignancy and other chronic diseases [26, 27]. In this patient population the spectra of infectious pathogens are largely not systematically studied. The knowledge of it will help clinicians make timely, evidenced-based decisions on antibiotic use before culture results return, which sometimes is life-saving [28–31].

With advanced machine learning techniques and inclusion of 13 parameters in the analysis, in current study we successfully developed four types of mathematical models to predict potential pathogens in patients with secondary immunodeficiency. Among these models, gradient Boosting Machine was found perform the best. To our knowledge, current study is the first systematically exploring the pathogen profile in patients with secondary immunodeficiency with mathematical models. In consideration of so many types of pathogens and practical clinical needs, we categorized the cultivated pathogens into seven species, which we think should be instructive for the empirical start of antibiotics therapy before culture results return.

While our study has provided valuable insights, it is important to acknowledge certain limitations in our methodological approach. In line with the PROBAST (Prediction model Risk Of Bias Assessment Tool) guidelines designed for evaluating the risk of bias and applicability of prediction model studies, we recognize that our use of 10-fold cross-validation for internal validation, though robust, may impart limitations in the context of external validation. Specifically, our model’s predictive performance could be overly optimistic if the training data are not representative of the broader population or future patients. Furthermore, despite the randomization process in cross-validation, unmeasured confounders and unknown biases inherent to the initial sample collection could still influence the results. PROBAST emphasizes the importance of validating prediction models in external datasets, originating from different settings or time periods compared to the data used for model development, to ensure applicability and generalizability. We have not yet had the opportunity to test our model on a completely independent external dataset, and such an external validation remains part of our future work. Until then, the generalizability of our findings should be considered with caution, as the chance of model overfitting to the idiosyncrasies of our dataset cannot be completely ruled out. We aim to address this by planning prospective studies that will challenge our model with diverse datasets across different clinical settings.

In addition, it was a single-center study and most of the data were from the hematology department. Therefore, the prediction models established in the current study need to be further verified in more patients with different secondary immunodeficiency from various geographic areas. We also think secondary immunodeficiency needs a more precise definition although it is truly hard to define since so many diseases can cause it, which poses challenges in standardization.

In conclusion, despite these limitations, our study presents models that we believe can assist clinicians, especially those from non-infectious disease departments, in making a timely evidenced-based decision regarding antibiotic use while awaiting culture results.

## Data Availability

All data generated or analysed during this study are included in this published article.
